# Biocompatible astaxanthin as a novel marine-oriented agent for dual chemo-photothermal therapy

**DOI:** 10.1371/journal.pone.0174687

**Published:** 2017-04-03

**Authors:** Van Phuc Nguyen, Sung Won Kim, Hanna Kim, Hyejin Kim, Kwang Hyuk Seok, Min Jung Jung, Yeh-chan Ahn, Hyun Wook Kang

**Affiliations:** 1 Interdisciplinary Program of Biomedical Mechanical & Electrical Engineering, Pukyong National University, Busan, South Korea; 2 Department of Otolaryngology-Head and Neck Surgery, Kosin University College of Medicine, Busan, South Korea; 3 Department of Biochemistry, Kosin University College of Medicine, Busan, South Korea; 4 Department of Pathology, Kosin University College of Medicine, Busan, South Korea; 5 Department of Biomedical Engineering and Center for Marine-Integrated Biomedical Technology (BK 21 Plus), Pukyong National University, Busan, South Korea; University of South Alabama Mitchell Cancer Institute, UNITED STATES

## Abstract

The photothermal effect of a marine-oriented xanthophyll carotenoid, astaxanthin (AXT), was characterized based on its potential absorption of visible laser light and conversion of optical light energy into heat for thermal treatment. As an antioxidant and anticancer agent, AXT extracted from marine material can be utilized for photothermal therapy due to its strong light absorption. The current study investigated the feasibility of the marine-based material AXT to increase the therapeutic efficacy of chemo-photothermal therapy (PTT) by assessing photothermal sessions in both cells and tumor tissues. A quasi-cw Q-switched 80 W 532 nm laser system was utilized to induce thermal necrosis in *in vitro* and *in vivo* models. An *in vitro* cytotoxicity study of AXT was implemented using squamous cell carcinoma (VX2) and macrophage (246.7) cell lines. *In vivo* PTT experiments were performed on 17 rabbits bearing VX2 tumors on their eyes that were treated with or without intratumoral injection of AXT at a dose of 100 *μl* (300 *μg/ml*) followed by laser irradiation at a low irradiance of 0.11 *W/cm*^*2*^. Fluorescence microscopy images revealed cellular death via apoptosis and necrosis owing to the dual chemo-photothermal effects induced by AXT. *In vivo* experimental results demonstrated that the AXT-assisted irradiation entailed a temperature increase by 30.4°C after tumor treatment for 4 min. The relative variations in tumor volume confirmed that the tumors treated with both AXT and laser irradiation completely disappeared 14 days after treatment, but the tumors treated under other conditions gradually grew. Due to selective light absorption, AXT-assisted laser treatment could be an effective thermal therapy for various drug-resistant cancers.

## Introduction

Current cancer therapies in clinical practice include surgery, chemotherapy, and radiation therapy [1–3]. Chemo- and radiation therapies often damage normal tissues while killing cancer cells, resulting in many side effects [4, 5]. Recently, thermotherapy has been widely investigated as a minimally invasive or non-invasive adjunctive therapeutic approach triggered by light [6, 7]. Photothermal therapy uses photoabsorbing agents to convert absorbed light energy into heat to kill cancer cells [8–10]. However, laser-based treatment can increase nonspecific injury in adjacent healthy tissue along the light path, as healthy tissue contains chromophores that also absorb laser light [11, 12]. To increase the speed and effectiveness of heat deposition to tumor cells while maintaining the temperature of surrounding tissue at a normal level, near infrared (NIR) absorbing dyes have been used to selectively increase the thermal destruction in target tumors [13]. Thus, avoiding damage to healthy neighboring tissues could be achieved by using photoabsorbing agents. Recently, nanotechnology research has engendered a number of inorganic nanomaterials, such as gold nanostructures [9, 14, 15], single-walled carbon nanotubes [16, 17], and copper sulfide nanoparticles [18], as promising photoabsorbing agents for photothermally coagulating or ablating cancer due to their strong near-infrared absorption efficiency. However, the major limitation of the abovementioned agents is their inorganic nature because their potential long-term toxicity remains unknown. Most of these agents also suffer from poor delivery and a small amount of the injected dose that is readily taken up by the target tissues [19].

To reduce toxicity in cancer treatment, a number of researchers have demonstrated the feasibility of eliminating tumors by modifying the surface of nanoparticles, such as biocompatible chitosan-coated gold nanoparticles and PEG-modified gold nanorods [10]. Recently, the use of organic materials as photothermal treatment (PTT) agents, such as indocyanine green (ICG), IR 780 dyes, porphysomes, polypyrroles and polymers [20–24], has attracted significant attention due to their low cytotoxicity. However, these agents have been shown to still be toxic to cells at high concentrations [25]. Furthermore, few studies have reported the optimal dosage and treatment conditions for completely removing the abnormal cells. It is arduous to deliver the optimal thermal energy to cancerous tumors due to the dependence of heat capacity on tumor characteristics such as type, stage, and size. Typically, cancer cells are more sensitive to heat than normal cells, and the temperature increases up to 42~47°C have been shown to induce apoptosis in cells [26], whereas the application of higher temperatures (i.e., 56°C or above) can lead to cancerous cell death directly through necrosis. Sultan *et al*. reported that the application of laser at high powers from tens to hundreds of watts efficiently induced tumor ablation [27]. However, the use of high light intensity can kill or weaken both normal cells and cancerous cells and eventually cause chronic inflammation during the healing process. Thus, to improve the effectiveness of PTT without inducing necrosis of normal cells, the applied laser power density should be reduced, and photoabsorbing agents should be applied at the appropriate dose. Jain *et al*. reported that the laser energy required for photodamage of cancer cells was 2~3-fold lower than that required for the nonmalignant cells (i.e., 19 W/cm^2^ for HOC malignant cells and 25 W/cm^2^ for HSC cancerous cells vs. 57 W/cm^2^ for healthy cells (HaCaT)) [12]. However, the high degree of laser irradiance still causes undesirable thermal injury to adjacent tissue. Therefore, reducing the amount of optical energy delivery is essential for developing a more effective drug-delivery system that has combined functions of both chemo- and photothermal therapy.

Astaxanthin (AXT) is a red-orange xanthophyll carotenoid pigment obtained from microalgae Haematococcus pluvialis, fungi, and marine organisms (i.e., crustaceans, salmon, shells of crabs, shrimps, and algae [28–30]), flamingos and quail [30]). Recent scientific literature has shown that AXT has considerable potential and promising applications in human health and nutrition [31]. The most significant activities of AXT include its antioxidative and anti-inflammatory properties, its effects on cancer, diabetes, and the immune system, and other related aspects. Fassett *et al*. showed that AXT has the potential to act as a therapeutic agent in atherosclerotic cardiovascular disease due to its potent antioxidant and anti-inflammatory properties [28]. Compared with inorganic therapeutic agents, AXT can provide a wide variety of benefits as a natural [32–34], non-toxic [35, 36] material that has a high molar extinction coefficient [37, 38]. Additionally, the United States Food and Drug Administration has approved AXT for use as a dietary supplement [31].

The main objectives of the current study were to address the feasibility of AXT as a novel photoabsorbing agent for multifunctional effects: enhancing heating during photothermal therapy (PTT) and improving anti-cancer activities on living rabbits with eye tumors. Upon illumination by 532 nm laser light, AXT can rapidly absorb laser light and convert the optical energy into heat to destroy cancer cells. The corresponding photothermal effects were evaluated in both cells and living animal models with eye tumors. In addition, a histological examination of the tumor tissues was conducted after thermal treatment via intratumoral injection of AXT to reveal pathological changes and to monitor tissue responses after the therapy.

## Materials and methods

### Chemical materials

All experiments were implemented with AXT extracted from *heamotococcus pluvialis* and purchased from Sigma-Aldrich Co. (St. Louis, MO, USA). Dimethyl sulfoxide (DMSO) was obtained from Samchun Pure Chemical Co., Ltd. (Pyeongteak-si, Gyeongi-do, Korea). All cell culture agents consisted of Dulbecco’s modified Eagle’s medium/F12 (DMEM-F12) obtained from Cellgro (Mediatech, Massachusetts, USA), fetal bovine serum (FBS), antibody, and phosphate-buffered saline (PBS) purchased from Gibco. 3-(4,5-dimethyl-2-thiazolyl)-2,5-diphenyl-2H-tetrazolium bromide (MTT) was obtained from Sigma-Aldrich. Hoechst 33342 and propidium iodide (PI) were obtained from Sigma-Aldrich. Distilled water was used for all aqueous solutions. All chemicals were directly used without further purification.

### Sample preparation

AXT was extracted from the green microalga *Heamotococcus pluvialis* as previously reported [39]. Prior to *in vitro* and *in vivo* experiments, 5 mg AXT was dissolved in 1 ml DMSO to prepare an AXT stock solution at a concentration of 5 mg/ml. For *in vitro* experiments on cancer cell lines, the AXT stock solution was diluted with DMEM/F12 medium to achieve the specified concentrations (i.e., 0, 100, 200, 300 μg/ml). For *in vivo* photothermal testing, the AXT stock solution was mixed with saline and stirred for 15 min to obtain a homogeneous distribution of AXT particles in the mixture.

### Characterization of AXT

The morphology of AXT was evaluated by using a scanning electron microscope (SEM) (Hitachi, S-2400, Japan). The SEM images revealed the distribution and estimated size of AXT particles. The mean size was analyzed by measuring the particle sizes of approximately 83 particles and plotting the corresponding histogram of particles size using ImageJ (National Institute of the Health, Bethesda, MD, USA). The absorption spectra of AXT was measured from 300 to 980 nm by using a spectrometer (XS2, BioTek, Winooski, VT, USA) to determine the suitable excitation wavelength for photothermal therapy.

To investigate the feasibility of AXT to induce a photothermal effect, four groups of AXT at concentrations of 0, 100, 200 and 300 μg/ml were prepared and poured into 96-well plates (total volume of 100 μl per well). Then, the samples were irradiated with a 532-nm laser system at an irradiance of 0.11 W/cm^2^. A 600-μm optical fiber (numerical aperture = 0.22) was utilized to deliver the laser light to the samples. The estimated laser beam size was 10.2 mm. The temperature rise during laser exposure was measured and recorded in real time by using an infrared thermal camera (FLIR A300, FLIR System, Inc., Sweden). The laser application time was set to 5 min for all experiments. Each experimental condition was repeated three times (N = 3).

### Cytotoxicity assessments

#### Cell culture

Squamous cell carcinoma (VX2) and macrophage cell lines (RAW 264.7) were purchased from the Korea cell line Bank (Seoul, Korea). RAW 246.7 cells were used to examine the toxicity of AXT solution on nonmalignant cancer cells. VX2 cells, which are epithelial cells originating from cancer, were selected to create tumors on the eyes of rabbits. Due to its relatively fast growth, this cell line is often used for small-medium animal models to evaluate preclinical cancer diagnosis and treatment [40]. These cell lines were used and cultured on monolayers in DMEM and DMEM-F12 (Thermo Scientific, Waltham, MA, USA), which were supplemented with 10% fetal bovine serum (FBS, Thermo Scientific, Waltham, MA, USA). The cultured cells were maintained at 37°C in a humidified atmosphere of 5% CO_2_ and 95% air. The cultured medium in the cell plates was changed three times per week. To harvest the cultured cells, a 0.025% trypsin-EDTA solution (total volume of 4 ml) was added to the wells and further incubated for 3 min.

#### Cell viability assay

To determine the degree of cytotoxicity of AXT, the prepared samples were tested with macrophage and VX2 tumor cancer cell lines. The VX2 cells were cultured in a 96-well microplate at a density of 1×10^4^ cells/well in 100 μl culture medium and allowed to grow for 24 h prior to treatment with AXT at different concentrations. A variety of AXT concentrations (0, 10, 20, 30, 40, 50, 100, 200, 300 μg/ml) were prepared and added to the cell culture medium. A cell culture with a blank medium (without astaxanthin) was used as a control. After treatment with AXT, the cells were further incubated at 37°C in a humidified atmosphere of 5% CO_2_ for 24 and 48 h to determine the effect of incubation time on cell survival. After various incubation times, the treated cells were supplemented with 50 μl medium and 50 μl MTT (1 μm/ml) reagent and were kept in a dark place to perform the cytotoxic analysis via an MTT assay. Then, the cells were incubated for 4 h. After incubation, the cells had changed to purple formazone crystals under microscopic inspection. Then, the supernatant was discarded, and 100 μl DMSO was added to each well. The 96-well plates were shaken gently for 20 min at room temperature to homogenize the color distribution prior to measuring the optical density (OD) of each well at 570 nm using an ELISA micro-plate reader (SpectraMax, 340, Molecular Device, Sunnyvale, CA, USA). The relative cell viability was calculated by using the following formula [41]:

$$P=\frac{OD\;value\;of\;experimental\;sample\;group}{OD\;value\;of\;control\;group}\;\times \;100$$ (1)

#### Cellular uptake

To evaluate interactions of AXT with cells, cellular uptake of the AXT solution was observed in terms of microscopic analysis. VX2 cells were seeded in 6-well plates at an estimated density of 4 × 10^5^ cells/well and incubated for 24 h at 37°C in a humidified atmosphere of 5% CO_2_. Then, the culture medium in the plates was discarded and replaced with fresh media containing 300 μg/ml AXT. The plates were further incubated for another 12, 24, and 48 h. For microscopic analysis, the cells were rinsed three times with cold PBS and fixed with 4% formaldehyde for 20 min at 37°C. Then, the plates were washed twice with PBS, and the cell morphology was observed by using a Leica optical microscope.

#### Effect of AXT on nonmalignant cells

Macrophage (264.7) cells were seeded in 96-well plates at an estimated density of 1 × 10^4^ cells/well and incubated for 24 h at 37°C in a humidified atmosphere of 5% CO_2_. Then, the culture medium was replaced with fresh medium containing AXT at various final concentrations (i.e., 0, 20, 30, 40, 50, 100, 200, and 300 μg/ml), and the cells were further incubated for 24 h. Cell survival was analyzed by an MTT assay.

#### In vitro photothermal cytotoxicity

To assess the photothermal cytotoxicity of AXT, VX2 cell lines were tested. The VX2 cells were seeded in 6-well plates at a density of 2 × 10^5^ cells/well and incubated for 24 h. Then, cultured cells were washed three times with cold PBS. Afterwards, the cells were treated with 2 ml AXT solution at the final concentration of 300 μg/ml and incubated for 4 h. The treated cells were then washed with PBS, and fresh media was added. Prior to laser treatment, the 96-well plates were placed inside a water bath maintained at 37°C. The cells were illuminated with visible laser light at an irradiance of 0.11 W/cm^2^ for 2–3 min. After laser treatment, the treated cells were stained with double-staining Hoechst 33342 and PI to examine the photothermal effect of AXT on cancer cells under a fluorescence microscope. To further investigate the photothermal cytotoxicity of AXT, VX2 cells (1 × 10^4^ cells/well) were seeded in the 96-well microplates and incubated for 24 h at 37°C in a humidified atmosphere of 5% CO_2_. Then, the medium in the plates was discarded and replaced with 100 μl fresh media containing the astaxanthin solution at the final concentration of 0 (control) or 300 μg/ml and further incubated for 4 h. After incubation, the cells were rinsed with cold PBS and irradiated with or without visible laser light at an irradiance of 0.11 W/cm^2^ for various times (i.e., irradiated time = 2 or 3 min). Following the laser application, the plates were further incubated for 12 and 24 h at 37°C in a humidified atmosphere of 5% CO_2_. The ratio of cell survival was observed by a standard MTT assay.

#### Apoptosis assay: Hoechst and PI double staining

Cell-permeable DNA dye Hoechst 33342 and propidium iodide (PI) were used to validate any changes in cell nuclear morphology, apoptosis, necrosis, and cell populations. The cells with the nuclei homogeneously stained were considered viable. The cells with the presence of chromatin condensation or fragmentation were indicative of apoptosis [42, 43]. On the other hand, cell necrosis was only stained with PI. VX2 cells were cultured in a 6-well plate at a density of 2 × 10^5^ cells/well and incubated for 24 h at 37°C in a humidified atmosphere of 5% CO_2_. The media were then replaced with fresh media containing AXT at the final concentration of 300 μg/ml, and the cells were further incubated for 4 h. The cells were washed three times with cold PBS prior to double staining with Hoechst 33342 and PI (i.e., DNA-specific fluorescent dyes). After washing, 300 μl Hoechst (10 μg/ml) was added to the cells, and cells were incubated at 37°C for 20 min. The cells were washed three times with PBS and supplemented with 300 μl propidium iodide (10 μg/ml). The plates were incubated for an additional 10 min at 37°C. The stained cells were washed three times and then visualized by using a Leica DMI3000B fluorescence microscope equipped with a DFC450C color digital camera (Leica, Wetzlar, Germany). To visualize the effect of the laser on the cells, the protocol was similar to the aforementioned method for studying the photothermal effect. At the end of the laser treatment, the cells were double stained with Hoechst and PI to determine the degree of nuclear condensation.

#### Statistical analysis

All experiments were performed three times, and the final values are presented as the mean ± standard deviation (SD). Statistical analyses of the data were performed by the Mann-Whitney U test using Statistical SPSS software package (Ver 22, IBM Corporation, Armonk, New York), and p < 0.05 was considered statistically significant (as shown in S1 Dataset).

### In vivo photothermal effect of AXT

All animal experimental procedures were implemented in accordance with the guidelines of the Korean National Institutes of Health (NIH). The protocol was approved by the Committee on Animal Research of the College of Medicine at Kosin University (Permit Number: KMAP-16-13). Seventeen New Zealand white rabbits, 10 males and 7 females, 3–4 months old, each weighing 2.2–2.6 kg, were purchased from Taesung Laboratory Animal Science (Busan, Korea) and used for *in vivo* experiments. All animals were housed in pathogen-free cages of the Animal Research Center facility and managed with *ad libitum* feeding. To create tumors on the eyes of rabbits, the animals were anesthetized with ketamine (35 mg/kg) and xylazine (5 mg/kg). The anesthetic depth was maintained via injections of 17.5 mg/kg/h ketamine and 2.5 mg/kg/h xylazine. Then, VX2 cells at a density of 1×10^7^ cells suspended in 40 μl PBS were subcutaneously injected into a subconjunctival area of each rabbit. The animals were carefully treated under the animal care protocol [44]. When the tumor progressed and reached a volume of approximately −50 mm^3^, tumor-bearing rabbit eyes were randomly classified into four groups for photothermal therapy: (1) a passive group (N = 6) with eye tumors that were intratumorally injected with 100 μl AXT dispersed in saline at a concentration of 300 μg/ml; (2) a blank control group (N = 4) with eyes treated with the same volume of saline without laser application; (3) a treatment group (N = 3) with eyes injected with 100 μl AXT at a concentration of 300 μg/ml followed by laser irradiation; and (4) a group treated with laser only (N = 4). The rabbits with and without injection of AXT were exposed with a quasi-continuous 532-nm laser system (pulse duration = 350 ns, repetition rate = 40 kHz, GreenLight PV^®^, American Medical Systems, Inc., San Jose, USA) at an irradiance of 0.11 W/cm^2^ as shown in S1 Fig. The laser beam size was estimated to be approximately 10.2 mm in dimeter, and the irradiation time was 4 min. The rabbits were anesthetized with an intraperitoneal injection of the ketamine and xylazine mixture prior to injection of the AXT solution. At 20 min after the injection, the rabbits were irradiated with the laser light. During the laser irradiation, the temperature development was recorded in real time by using a digital IR thermal camera. The thermal camera was switched on simultaneously with the onset of laser exposure. All the animals were treated only one time. The size of the tumors (i.e., length (l) and width (w)) was measured by using calipers every day after the laser irradiation. The tumor volume was calculated by using the following formula [45–47]:

$$volume=\frac{1}{2}\times tumor\;length\;\times \;{\left(tumor\;width\right)}^{2}$$ (2)

In addition, the tumor growth inhibitory rate (%TGI) was determined after 14 days by using the following equation [45, 48]:

$$\%TGI=100\%\times \frac{\left(T_{c}-T_{t}\right)}{T_{c}}$$ (3)

where T_c_ and T_t_ represent the mean tumor volume of the control and treated groups, respectively.

### Histological assessment

To reveal the potential of *in vivo* toxicity of astaxanthin, rabbits with eye tumors after PPT application were euthanized by overdose with CO_2_ gas, and the tumors were extracted for histological examination after 6 days of treatment. All tumor tissues from the control and treated groups were fixed in 10% neutral buffered formalin and embedded in paraffin for standard hematoxylin & eosin (H&E) staining. Each sample was cross-sectionally cut in thicknesses of 4 μm, and all sections were simultaneously stained with hematoxylin and eosin (H&E) using a Leica autostainer XL (Leica Biosystems, Nussloch, Germany). The slides were then examined using an Olympus BX51 light microscope (Olympus Corp., Tokyo, Japan).

## Results and discussion

### Characterization of AXT

**Fig 1:**
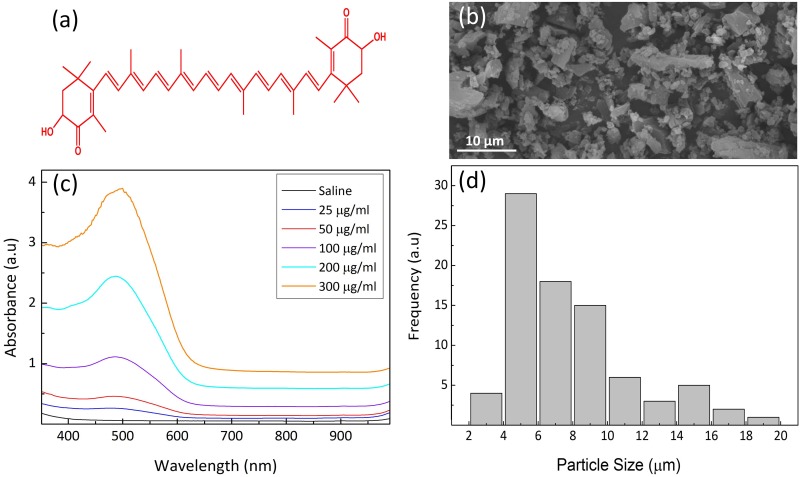
Characterization of Astaxanthin (AXT). (a) Chemical structure, (b) TEM image (×2000, 20 kV), (c) absorbance as a function of wavelength and concentration, and (d) distribution of AXT particles sizes. Note that the average particle size was measured to be 7.9 ± 3.6 μm.

Fig 1a presents the chemical structure of AXT utilized for the current study. The scanning electron microscope (SEM) image of AXT shown in Fig 1b demonstrates the surface morphology of AXT particles. Based upon the SEM image, the minimum and maximum sizes of the AXT particles were quantitatively evaluated in Fig 1d. The AXT particle size was estimated to be approximately 2.93 ± 0.23 to 18.90 ± 0.29 μm. In addition, Anarjan *et al*. showed that the average particle size of an AXT dispersion is approximately 115~ 122 nm, which is suitable for the delivery of drugs to tumors through the enhanced permeability and retention effect (EPR) [49, 50]. In fact, the size of particles plays an important role in the performance of the drug as a photothermal agent. Huang *et al*. reported that small nanoparticles are preferable for photothermal therapy because light is mainly adsorbed by the particles and, thus, is efficiently converted into heat for cell and tissue destruction [51]. However, particles smaller than ~10 nm are easily cleared from systemic circulation via the kidneys [52]. Fig 1c shows the absorbance spectra of AXT in saline as a solvent. Although the maximum absorption of AXT is dependent on the refractive index of the solvents [37], the spectral measurements showed that strong absorption bands of AXT occurred in the visible wavelengths ranging from 480 to 540 nm. It was noted that the absorption of AXT (i.e., 300 μg/ml) was approximately 67-fold higher than that of saline (i.e., absorbance = 0.06 for saline vs. 3.89 for AXT at a concentration of 300 μg/ml). Accordingly, the wavelength of 532 nm was selected as the commercially feasible excitation wavelength for PTT.

### Effect of AXT on nonmalignant cells

**Fig 2:**
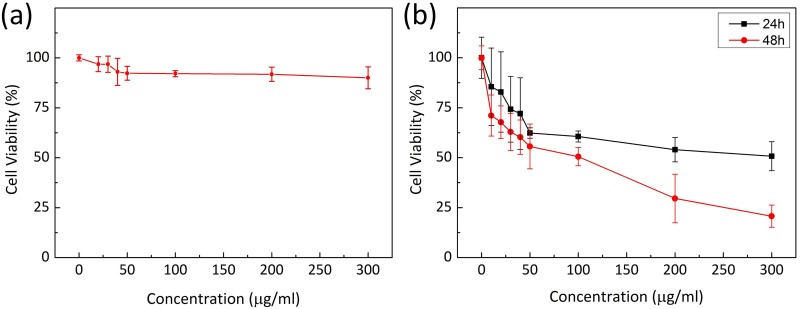
Cytotoxicity of AXT. (a) Effect of AXT on macrophage (RAW 264.7) cells after 24 h and (b) effect of AXT on VX2 cells at various concentrations (0, 10, 20, 30, 40, 50, 100, 200, 300 μg/ml) and incubation times (24 and 48 h). Each error bar represents one standard deviation.

The cytotoxicity of AXT was evaluated on macrophage cell lines. The cell viability results shown in Fig 2a evidenced that AXT is non-toxic to nonmalignant 246.7 cells, unlike other nonorganic agents, such as gold nanoparticles or single-walled nanotubes [53, 54]. The survival rate of the treated macrophage cells slightly decreased compared to untreated control cells as the concentration of AXT increased (i.e., survival rate = 96.8, 96.8, 93.0, 92.3, 92.1, 91.8, and 90.0% for 20, 30, 40, 50, 100, 200, and 300 μg/ml AXT, respectively). At the highest concentration of AXT (i.e., 300 μg/ml), the cell survival was still 90.0%, implicating no significant toxic effect 24 h after AXT application. The persistent survival rate of the cells treated with AXT even at high concentrations indicates the non-toxic and biocompatible properties of AXT [30]. However, a limitation of the current study is that cellular toxicity of AXT was only tested in 246.7 cells. Therefore, future studies will be conducted on other types of normal cells to further elucidate the cytotoxic effect of AXT. Recently, AXT has been acknowledged by the FDA as a nutraceutical and is being developed for pharmaceutical applications in healthcare, including applications in prostate and cardiovascular cancers, hepatitis, Alzheimer’s disease, and age-related macular degeneration [30, 31]. Extensive studies have evaluated the pharmacological and toxicological safety of AXT in both animals (i.e., rats and rabbits) and humans [34, 55]. These studies revealed no evidence of AXT toxicity at doses of up to 1000 mg/kg in animal models.

### Cytotoxicity evaluation

*In vitro* cell viability experiments were performed to identify the anti-cancer effects of AXT on tumor cells. Fig 2b presents the cytotoxicity effects of AXT on squamous cell carcinoma (VX2) as a function of AXT concentration and incubation time. Overall, the cell viability decreased with both concentration and incubation time. For an incubation time of 24 h, a rapid decrease in cell viability was initially observed with increasing concentrations, but after 100 μg/ml, the change in the viability became saturated. On the other hand, longer incubation times (48 h) yielded a continuous decrease in cell viability with increasing AXT concentrations. The controls exhibited no change in cell viability for all incubation times. At the highest concentration of AXT (300 μg/ml), the rate of cell survival was approximately 50% after 24 h of incubation. However, the majority of cells were killed as the incubation time increased to 48 h, corresponding to almost 75% cell death compared with the control. The experimental results clearly evidenced the excellent anticancer activity of AXT against VX2 cells, which agrees well with the previous findings [56].

**Fig 3:**
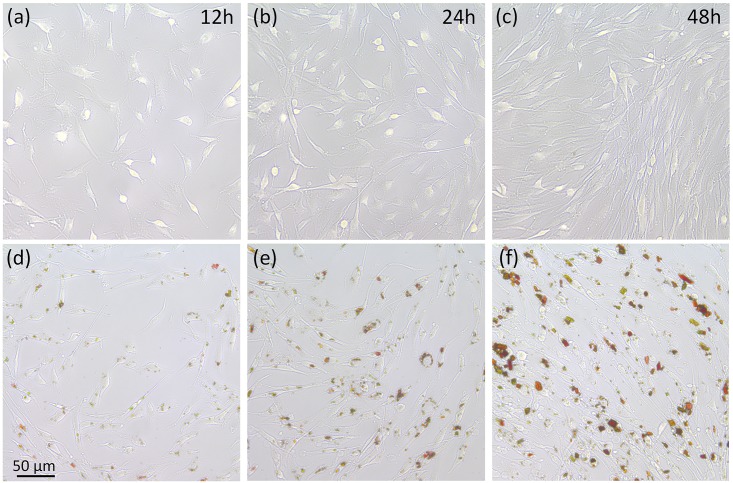
Cellular uptake of AXT at different incubation times. (a-c) Untreated cells after incubation for 12, 24, and 48 h, respectively, and (d-f) cells treated with AXT at a concentration 300 μg/ml and incubation times of 12, 24, and 48 h, respectively.

To achieve a desirable therapeutic effect, AXT needs to be taken up through cell membranes. VX2 cells were cultivated with AXT at a concentration of 300 μg/ml and incubated for different times (i.e., 12, 24 and 48 h). Then, the cellular uptake of AXT was observed under a digital confocal microscope. Fig 3 shows the intracellular uptake of AXT solution. Red color represents the presence of AXT in Fig 3d and 3e, whereas no color was observed for cells incubated without AXT in Fig 3a–3c. It was noted that AXT gradually accumulated in cells as the incubation time increased. In addition, the cells also proliferated with increased incubation time. According to the cell morphology by AXT staining, excellent cellular uptake occurred at the incubation time of 48 h (Fig 3f), implicating that AXT may have the potential to treat tumor cells in terms of anti-tumor drug delivery.

### Photothermal effect of AXT

**Fig 4:**
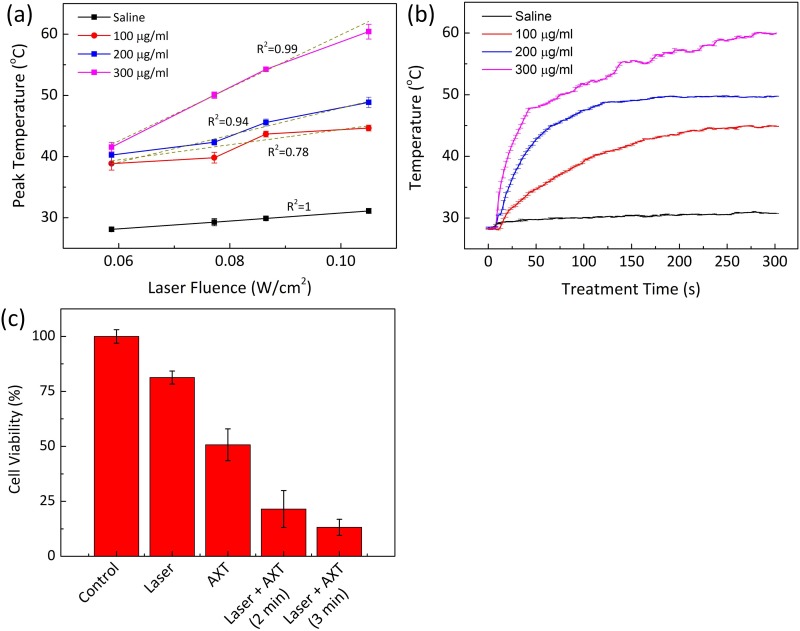
In vitro PTT effects. (a) Comparison of peak temperature changes at various AXT concentrations as a function of laser irradiance, (b) temporal development of temperature at concentrations of 0 (saline), 100, 200, and 300 μg/ml AXT in 1 ml aqueous solution (laser irradiance = 0.11 W/cm2), and (c) quantified cell viability under various testing conditions: control, laser irradiation only, AXT only, and AXT-assisted laser treatment for 2 and 3 min (300 μg/ml of AXT). Data are expressed as the mean ± standard deviation (N = 3).

To verify the feasibility of AXT as a photothermal agent, both saline and AXT were illuminated with 532-nm laser light. Fig 4a demonstrates the peak temperature response of the samples as a function of laser irradiance for 5 min of irradiation. Overall, the peak temperature almost linearly increased with laser irradiance and AXT concentration. According to Fig 4a, the lowest AXT concentration (100 μg/ml) increased the temperature from 38.8 ± 1.1°C at 0.06 W/cm^2^ (transient temperature change = 0.04°C/s) up to 44.7 ± 0.5°C at 0.11 W/cm^2^ (0.06°C/s) after 5 min of irradiation. The highest concentration (300 μg/ml) induced a peak temperature that varied from 41.5 ± 0.8°C at 0.06 W/cm^2^ (0.05°C/s) up to 60.4 ± 1.2°C at 0.11 W/cm^2^ (0.11°C/s). On the other hand, saline corresponded to only a slight temperature elevation (from 28.1 ± 0.4°C to 31.1 ± 0.4°C) due to insignificant light absorption at 532 nm. As the temperature threshold for irreversible thermal denaturation corresponds to 55 ~ 95°C [57], a laser power density of 0.11 W/cm^2^ and an AXT concentration of 300 μg/ml were selected and used as the appropriate experimental conditions for the remaining *in vitro and in vivo* tests. Previous studies have shown that laser irradiance of approximately 433 W/cm^2^ can induce the required temperature increase for tissue denaturation (> 55°C) [58], which often damages normal tissue around the targeted tumors [59]. In contrast, the current study demonstrated the feasible integrating AXT with laser irradiation that could allow for the application of low-laser irradiance (i.e., 0.11 W/cm^2^) for thermal treatment and minimize the risk of thermal injury to adjacent tissue [60]. Fig 4b demonstrates the temporal development of temperature during laser irradiation of aqueous AXT solution at the irradiance of 0.11 W/cm^2^ for 5 min. Overall, the temperature rapidly increased with AXT concentration and irradiation time but became saturated possibly when thermal equilibrium was reached. Under identical irradiation conditions, the temperature of AXT solutions increased upon irradiation by laser light from the initial temperature (T_0_ = 27°C) to 44.7 ± 0.5, 48.9 ± 0.9, and 60.4 ± 1.2°C for 100, 200, and 300 μg/ml, respectively. AXT at the highest concentration (i.e., 300 μg/ml) induced a temperature rise up to 60.4°C, which was up to 1.4-fold higher than the temperature rise at lower concentrations.

To evaluate the phototherapeutic effect of AXT on VX2 cells, the degree of cell death was investigated by determining cell viability after laser exposure via an MTT assay. Fig 4c presents the cytotoxic effect resulting from photothermal application. Generally, the rate of cell survival decreased with the total irradiation time. The higher percentage of the cell viability upon laser irradiation only indicated no adverse effect from the laser light on the cancer cells (i.e., 81% for 24 h of incubation). In contrast, the cytotoxic effect was enhanced when laser irradiation was performed with the AXT solution. Compared to the laser only, the AXT-assisted laser irradiation corresponded to 2.7-fold lower cell viability (i.e., 88% for laser vs. 33% for combined treatment). Under the same treatment conditions (i.e., concentration = 300 μg/ml and irradiance = 0.11 W/cm^2^), the increase in laser irradiation led to a higher rate of cell death (79% for 2 min vs. 87% for 3 min). In addition, compared with the group treated with AXT (300 μg/ml and incubation time for 24 h), AXT-assisted laser treatment achieved 37% higher cellular death (i.e., 50% for the group treated with AXT vs. 87% for the group treated with AXT and laser exposure for 3 min). The proportion of cells was reduced after treatment with laser light, indicating that the treatment efficacy was enhanced by the introduction of AXT and PTT.

**Fig 5:**
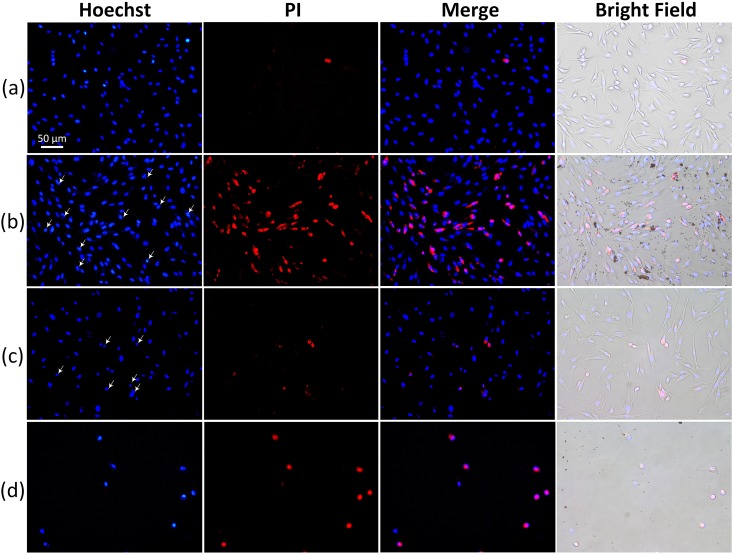
Double staining of VX2 cells with Hoechst and PI. (a) control (untreated cells without AXT solution), (b) cells treated with AXT (300 μg/ml), (c) cells exposed to laser only (532 nm, 0.11 W/cm^2^, and 3 min), and (d) cells treated with AXT (300 μg/ml) and exposed to laser (532 nm, 0.11 W/cm^2^, and 3 min). Nuclei were stained with Hoechst 33342 (blue). Dead cells were stained by using PI and are shown in red (Magnification = 20× and scale bar = 50 μm).

To evaluate anti-cancer performance and the efficacy of AXT-assisted PTT, *in vitro* cell experiments were performed and qualitatively evaluated. The application of laser light can induce diverse cancer cell death via apoptosis and necrosis, which are dependent on the irradiation time [61]. Typically, apoptotic and necrotic cell death represent variations in nuclear morphology, such as chromatin condensation, fragmentation, cell shrinkage, and blebbing of the plasma membrane in the nucleus [62]. Consequently, morphological changes were observed by using a digital optical microscope. In this study, VX2 cells were incubated with aqueous astaxanthin at a concentration of 300 μg/ml for 3 h. Then, the AXT-treated cells were irradiated with 532-nm laser at an irradiance of 0.11 W/cm^2^ for 3 min. After irradiation, the cells were stained with Hoechst 33342 and propidium diode (PI). Fig 5 shows the morphological changes of VX2 cells observed by using a fluorescence microscope. The blue emission came from the Hoechst that stained the nuclei of viable VX2 cells. The red cells represent dead cells resulting from the PI staining. It was found that both control and laser-only treated samples displayed a few of the marked changes in cellular morphology (PI staining) and a clear contour of nuclei, indicating that the cells were viable. Thus, the cells treated with and without laser irradiation exhibited insignificant cytotoxicity. However, the groups of cells incubated with AXT and irradiated with laser light presented both apoptotic (stained by Hoechst 33342) and necrotic (stained by PI) cellular responses, suggesting that cellular death occurs via both apoptosis and necrosis. The vividly decreased viability of VX2 cells as shown in Fig 5d evidenced the feasible enhancement of anticancer effects resulting from a combination of AXT and PTT on the cancer cells.

**Fig 6:**
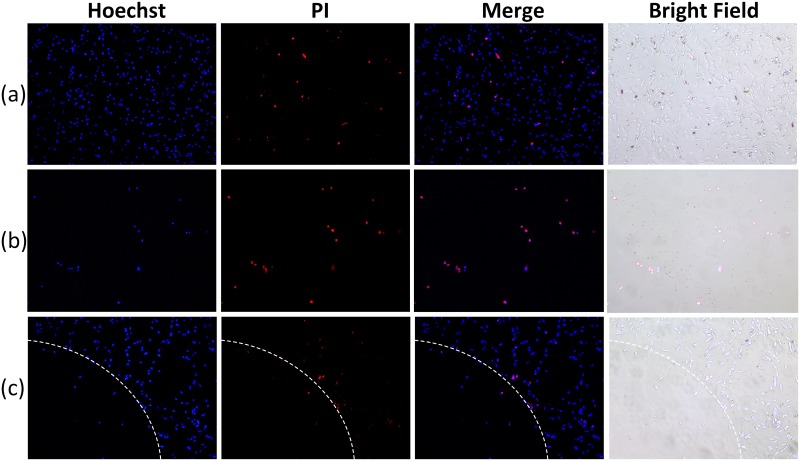
Fluorescence microscopy images of VX2 cells after irradiation with 532-nm laser light at different positions and double staining with Hoechst and PI. **(a)**. Fluorescence and bright field images of cells from (a) outside, (b) inside, and (c) on the edge of the treated area (Magnification = 20× and scale bar = 50 μm).

Fig 6 shows fluorescence images of VX2 cells located (a) outside, (b) at the center, and (c) on the edge of the laser-treated region. Most cells located outside the treated region of the laser were viable, as shown in Fig 6, indicating the selective targeting and the safety of the laser treatment. However, all the cells in the treated region were dead due to the photothermolytic effect of AXT (Fig 6b). Fig 6c demonstrates a clear boundary between the irradiated and non-irradiated areas. It should be noted that the cells along the boundary were dead, indicating that cellular death after AXT uptake was contingent on the laser irradiation.

### In vivo photothermal therapy

**Fig 7:**
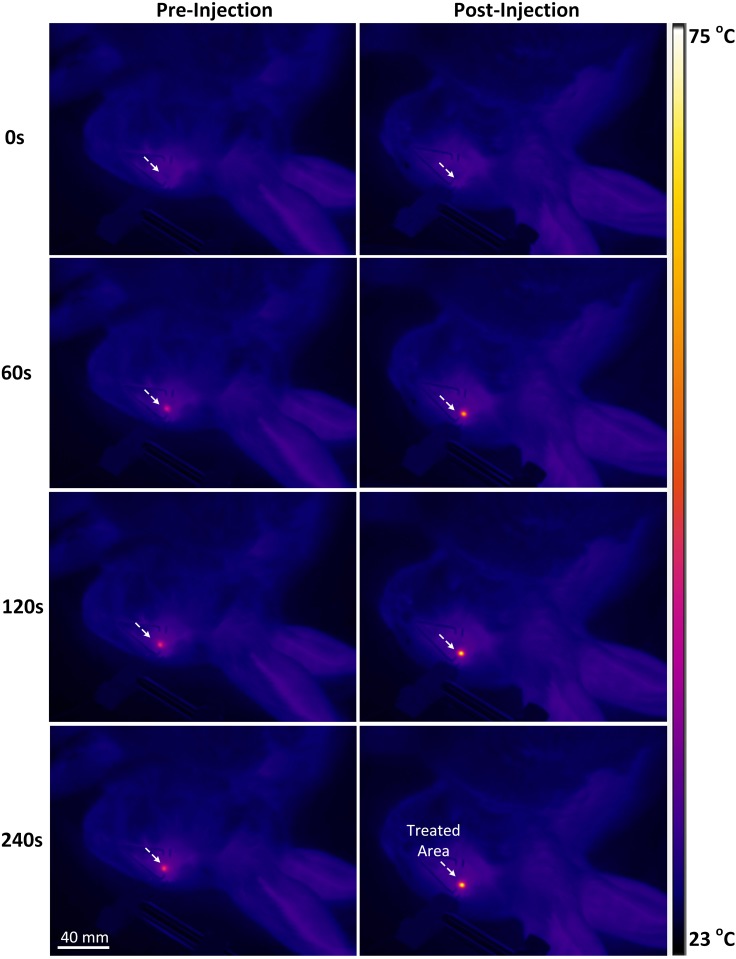
IR thermal images of eye tumors treated without (no injection) or with AXT (injection) followed by irradiation with 532-nm laser light (irradiance = 0.11 W/cm^2^) at various irradiation times up to 240 s.

*In vivo* AXT-assisted photothermal therapy was evaluated in VX2 tumor-bearing white rabbits. When each tumor volume reached approximately 50 mm^3^, the eye tumors in each rabbit were intratumorally injected with the AXT suspension at a dose of 100 μl (300 μg/ml). Twenty minutes after injection, each eye tumor was irradiated with 532-nm laser light at an irradiance of 0.11 W/cm^2^ for 4 min. Fig 7 shows the spatio-temporal development of the temperature of the eye tumor imaged by using a digital IR thermal camera (FLIR A300, FLIR system, Sweden) during the photothermal treatment. White arrows indicate the position of the treated areas. During the laser application, animals in groups both with and without AXT injection presented a temperature increase. However, the AXT-injected group experienced up to 24% higher temperature increase than the group that did not receive injections (54.7°C for injection vs. 67.6°C for non-injection at 240 s of irradiation).

**Fig 8:**
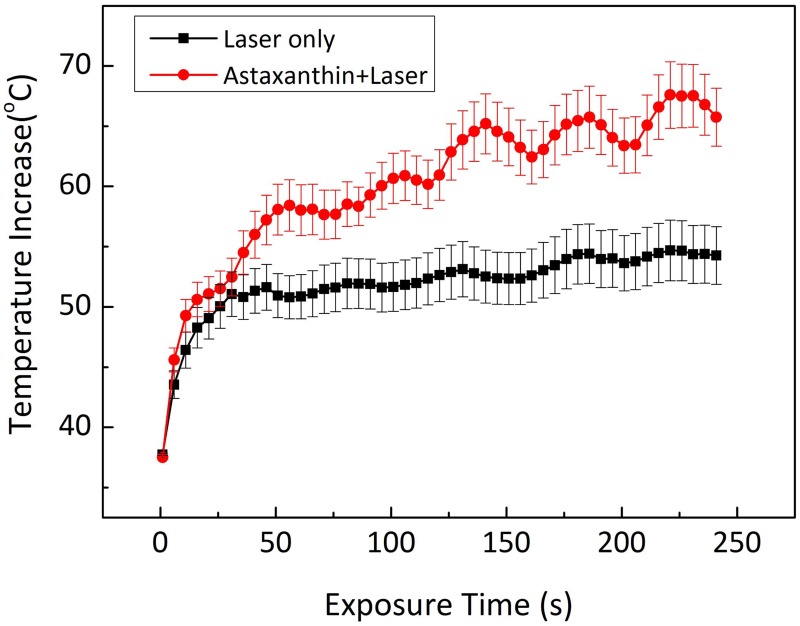
Temporal development of temperature in the tumor area during laser irradiation without or with AXT.

Fig 8 presents the temporal developments of temperature in the rabbit eye tumors during laser irradiation. The temperature of the tumors treated with the laser only (no AXT injection) exhibited a temperature increase from 37.0±0.1°C to 54.7±2.5°C after 4 min of irradiation. On the other hand, the tumors treated with AXT and laser light exhibited a significant temperature elevation up to 67.6± 2.8°C under the same condition, which was 25% higher than the temperature of tumors for the laser-only condition and sufficient to eliminate tumors cells [45]. In fact, under the same irradiation conditions, the temperature of the tumors treated without AXT exhibited almost no changes in the treated area compared to the AXT-assisted laser treatment. Thus, the current results demonstrate the successful use of AXT for enhancing the heating effect of laser application and to eventually induce complete irreversible tissue damage [63] [64]. It was also noted that the irradiance of 0.11 W/cm^2^ was enough to effectively destroy the tumors *in vivo*, which was much lower than that from other laser wavelengths combined with various photothermal dyes [58]. The improved energy coupling efficiency could result from the strong light absorption of AXT at the wavelength of 532 nm. The current study selected the commercially available wavelength of 532 nm for AXT-assisted PTT due to the availability of such laser systems. Moreover, the peak absorbance wavelength of AXT occurred at 532 nm and, therefore, might be suitable for the treatment of superficial tumors as the optical penetration depth of the 532-nm laser was limited to 1~2 mm [65, 66]. However, both hemoglobin and AXT exhibit strong light absorption/scattering in visible light ranging from 480 to 540 nm, which might adversely damage blood vessels or adjacent tissue during laser exposure [67]. To achieve deeper tissue treatment and to avoid undesirable damage to adjacent tissues, another excitation wavelength in the near-infrared range should be investigated. For instance, Raja *et al*. reported that AXT conjugated with AuNPs could generate two absorption peaks at 532 and 985 nm [68]. Thus, the wavelength of 985 nm could be used for both therapeutic and diagnostic applications for deep tissue in the body with promising efficiency. Although various benefits of AXT on human health, such as immunomodulatory, anti-stress, anti-cancer, and anti-inflammatory properties in a number of pathological indications [35, 56, 69], have been suggested, the use of AXT for PTT was previously studied in cells by using AXT as derivative agent to reduce the toxicity of nanoparticles [68]. Thus, the current study experimentally demonstrated the feasible application of AXT as a promising photothermal agent along with its easy fabrication and favorable pharmacokinetics [30].

**Fig 9:**
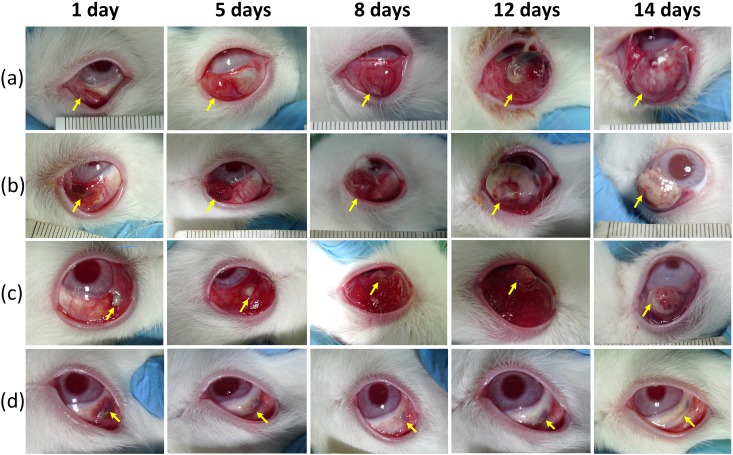
Gross images of tumor-bearing rabbit eyes after *in vivo* AXT-assisted photothermal treatment. (a) Rabbit eye xenografted with VX2 tumor cells, (b) tumors treated with laser (532 nm, 0.11 W/cm^2^, and 4 min), (c) tumors treated with AXT solution (300 μg/ml) intratumorally injected without laser treatment, and (d) tumors treated with both AXT and laser. No abnormal behaviors were observed in rabbits 14 days after each treatment.

Fig 9 shows further investigations of the chronic response of tumors after photothermal treatment with 100 μl (i.e., 300 μg/ml) AXT injection for various testing conditions: (a) control, (b) tumors treated with laser only, (c) tumors treated with AXT only, and (d) tumors treated with AXT and laser together. AXT solution (i.e., 300 μg/ml) was injected into rabbits with eye tumors. Then, the animals were exposed to a laser beam at a power density of 0.11 W/cm^2^ for 4 min. To avoid any tissue damage due to heating, the laser application was interrupted at each interval of 1 min laser exposure. After the laser treatment, the tumor growth rate was evaluated for all groups of animals including the untreated rabbits (control, N = 4), rabbits irradiated with the 532-nm laser light (laser only, N = 4), and AXT-injected rabbits with and without laser application (N = 6 for non-laser irradiation and N = 3 for laser irradiation) every day for 14 days. The control group clearly showed continuous and significant tumor growth (approximately 1 cm in diameter) after 14 days in Fig 9a. In the case of the group treated with AXT only, as shown in Fig 9b, the tumor continuously grew, but the increase in tumor size was slightly smaller (approximately 0.8 cm) than that of the control group. Inhibition of tumor growth by the natural marine material could contribute to the decreased tumor size, which is consistent with the accumulation of AXT in tumor tissues [56, 70]. The group treated with laser only in Fig 9c initially showed acute necrotic tissue (discoloration) due to photothermal coagulation at day 1, but the tumor size still increased up to approximately 0.8 cm by 14 days after the laser treatment. According to Fig 9d, the animals treated with both AXT and laser displayed marked injury with tissue denaturation at the irradiated region. The size of the tumor shown in Fig 9d was substantially decreased and became markedly smaller with time compared to the other groups (Fig 9a–9c). At day 14, the group treated with the combination of AXT and laser irradiation demonstrated almost complete elimination of the tumor. The necrotic scar tissue was almost completely healed by 6 days following the laser application, and normal tissue began to regenerate in the treated areas.

**Fig 10:**
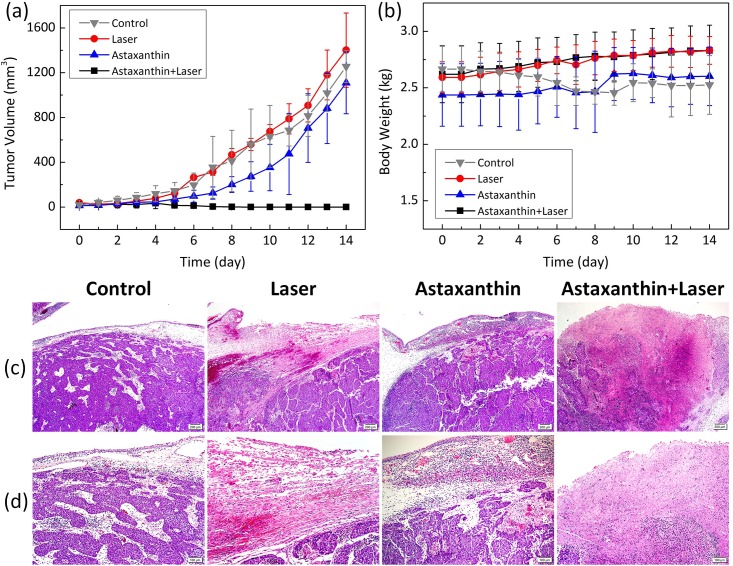
Antitumoral activity of AXT-assisted laser-induced photothermal therapy in tumor-bearing rabbit eyes. (a) Growth of VX2 tumors and (b) body weight of various groups after treatment as a function of time. The tumor volume was recorded every day for 14 days, and data are expressed as the mean ± standard deviation. H&E-stained images of tumors from various groups (control, laser, AXT, and AXT-assisted laser): (c) ×40 and (d) ×100.

Fig 10 presents the quantitative evaluations of (a) tumor volume and (b) animal body weight for the 14 days following treatment. Overall, the estimated volume of solid tumors initially showed slow tumor growth (approximately 50 mm^3^) but rapidly increased after 6 days up to approximately 1300 mm^3^ at day 14 for the control, AXT, and laser-only groups (Fig 10a). In contrast, the group treated with both AXT and laser demonstrated complete elimination of tumor tissue (i.e., variations in tumor volume from 50 to 0 mm^3^) over 14 days after the combined treatment, and the tumor growth inhibitory ratio was approximately 100% compared with controls. The tumor volume of the group treated with laser irradiation alone was almost equivalent to that of the control group, indicating that application of laser yields minimal therapeutic effects on tumorous tissue, which well agrees with the previous study [45]. In fact, laser treatment with low irradiance would expect to induce no collateral tissue injury [15, 71]. On the other hand, all the tumors treated with the combination of AXT and laser irradiation disappeared and yielded no recurrence after two weeks of follow-up. Therefore, AXT could be used as a feasible photothermal agent for *in vivo* PTT treatment of tumors. To investigate any side effects and potential toxicity of AXT application, the body weights of all treated animals were monitored every day. Body weight is commonly used to evaluate the health of treated animals [21]. Loss of body weight is associated with treatment-induced toxicity. Fig 10b shows that the body weight of the rabbits gradually increased during the 14 days after various treatment conditions, implying that systemic toxicity was minimal in all of the tested groups. Accordingly, *in vivo* AXT-assisted photothermal therapy was associated with a vivid inhibitory effect on tumor growth with minimal adverse effects and complications [21], indicative of the potential natural adjuvant for thermal treatment.

### Histopathological analysis

To assess the efficacy of laser treatment on rabbits with eye tumors, histological analysis of the tumors was performed for the various treatment groups 14 days after treatment, as shown in Fig 10c and 10d. Tumors treated with saline buffer showed minimal necrotic change in the intratumoral or superficial portion of the residual tumor, which was similar to the groups treated with laser and AXT alone. In marked contrast, the tumors treated with the combination of AXT and laser exhibited wider and deeper necrotic changes, which had a wedge-shaped orientation based on a superficial portion of the residual tumor and corresponded to a discolored region upon gross examination. In addition, complete regression of tumor cells and marked infiltration of inflammatory cells, such as foamy macrophages, lymphocytes, and eosinophils, were also observed in the tissues treated with the combination treatment. Thus, the histological analysis confirmed that complete thermal therapy was achieved with the AXT-assisted laser thermal treatment. These results clearly demonstrate the potential clinical application of AXT as a PTT agent. However, further studies are still needed to systematically analyze the potential long-term toxicity of AXT at various doses in animals.

## Conclusion

The current study demonstrates the potential dual chemo-photothermal effects of the marine-derived material AXT to treat eye tumors at low laser irradiance *in vitro* and *in vivo*. Due to its strong and selective light absorption at 532 nm, biocompatible AXT can enhance thermal effects during laser irradiation to destroy cancerous tumors and achieve complete tumor eradication without any significant toxic effects. Further investigations are underway to achieve specific cancer targeting of AXT-based agents via intravenous administration. The marine-derived AXT may be a potential biocompatible photothermal adjuvant for effective cancer treatment.

## Supporting information

S1 Fig Experiment setup for photothermal therapy. (TIF)

S1 Dataset Statistical analysis. (XLSX)
