# Supplementary figures and images for: Biocompatible astaxanthin as a novel marine-oriented agent for dual chemo-photothermal therapy
Source: PLoS One. 2017 Apr 3;12(4):e0174687. doi: 10.1371/journal.pone.0174687 (PMC5378353; doi:10.1371/journal.pone.0174687)

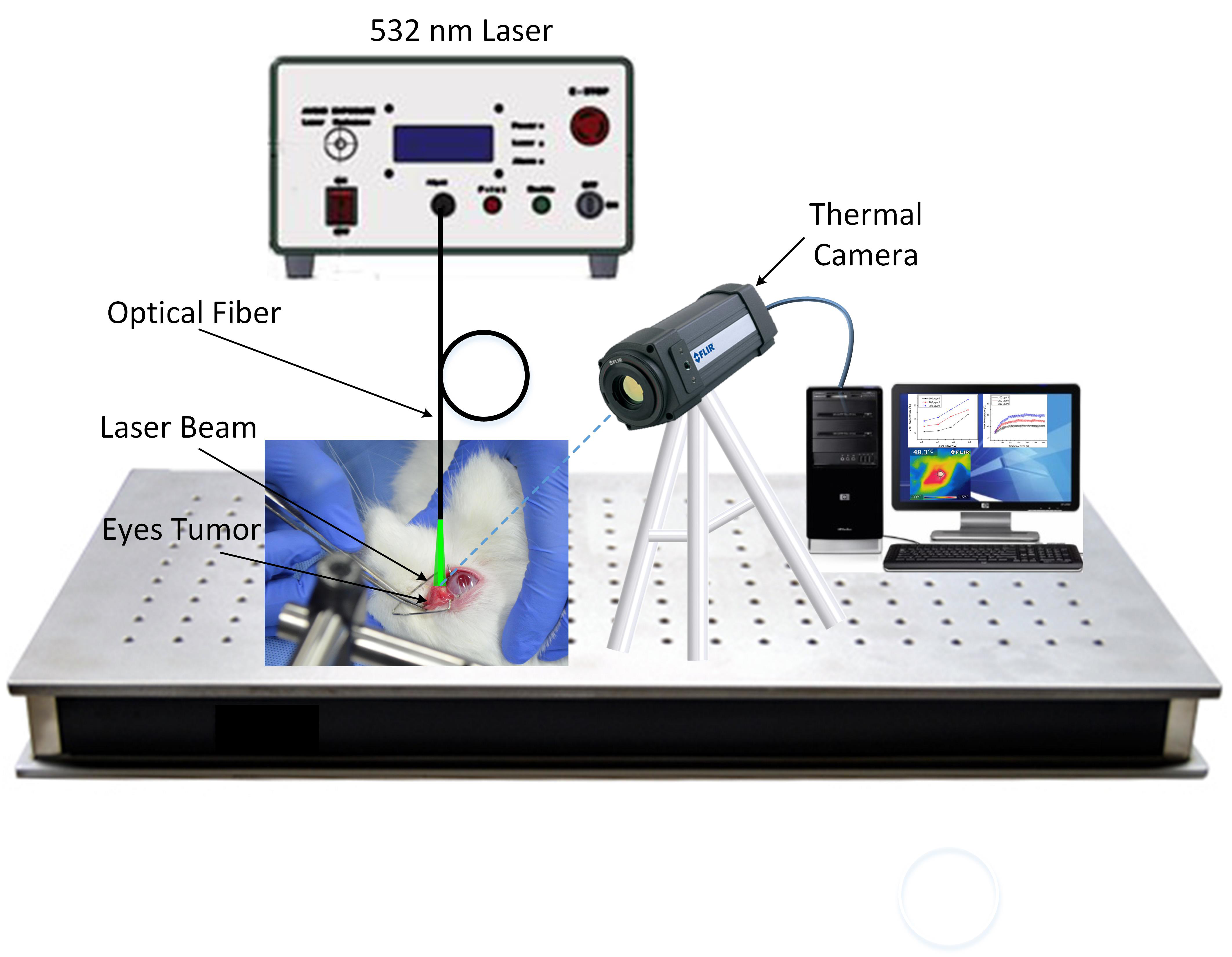

Supplement: S1 Fig — (TIF) [file pone.0174687.s001.tif]
